# Massachusetts—Prevention and Eradication of Bovine Tuberculosis

**Published:** 1894-12

**Authors:** Alex. Burr, Jas. B. Paige, John M. Parker

**Affiliations:** Committee on Tuberculosis; Committee on Tuberculosis; Committee on Tuberculosis


### MASSACHUSETTS.

Realizing the importance of preventing the extension and continuance of bovine tuberculosis among our dairy herds, the Massachusetts Veterinary Association has prepared the following brochure in the hope that it may assist in spreading information on the prevention and eradication of this disease.

In referring to tuberculosis, the following questions are often asked by stock owners: (1st) how shall the occurrence of tuberculosis be prevented in a healthy herd, and (2d) how shall tuberculosis be eradicated from a herd that is already diseased.

In considering these questions it should be borne in mind that while it is a fact that, no matter how unhealthy the surroundings, bovine tuberculosis cannot exist without the presence of the bacillus, yet it is equally a fact that the germ requires a suitable soil for its development, and that a favorable condition of the body for the development of tuberculosis frequently results from hereditary predisposition, unsanitary surroundings and the injudicious management to which dairy cattle are so often subjected. It follows then that anything that tends to undermine the health of dairy stock should be avoided, and a continual effort made to strengthen and build up the constitution of the dairy cow.

The following recommendations are made to stock owners, to prevent the occurence of tuberculosis in a healthy herd:

1. As far as possible owners should raise their own stock, and endeavor to improve the constitution of the herd by breeding only from animals that are strong constitutionally and known to be free from any tuberculosis taint.

2. When practicable, all farmers should own a bull. They should restrict its use to their own cows and not allow it to come in contact with other stock.

3. Allow no strange animal to come in contact with the herd without first making sure, by tuberculin test (which is now recognized to be the only practical method of diagnosis,) that they are free from disease.

4. Never buy from infected or suspicious herds.

5. Never purchase a cow with a cough or abnormal breathing, lumpy or diseased udder, swollen joints, or with a tendency to scour or bloat.

6. Overcrowding in barns should be avoided. Provide as much air space as possible, allowing at least 1,000 cubic feet for each animal.

7. Pure air and abundant sunlight are essential to the preservation of health in animals. Windows hinged at the bottom and dropped slightly inward at the top may be utilized for light and ventilization. In this way the air is directed upward, thereby preventing a current of cold air on the cattle.

8. In fair weather cattle should be in the open air as much as possible.

9. All barns should be kept as clean as possible, they should be sprinkled before being swept, and in consequence of the irritating and infectious character of the dust of stables, in which tuberculosis animals have been kept, sweeping should always be done while the cattle are in the yard.

10. In consequence of the danger to cattle from consumptives expectorating in and around barns, no consumptive person should be allowed to have charge of or come in contact with dairy cattle.

11. Do not keep manure in the cellar. Better have no cellar, but where one exists it should be well drained, well lighted and well ventilated.

12. Manure should be frequently removed from the neigborhood of barns.

13. The barnyard and its surroundings should be well drained and free from standing water and filth.

14. Early breeding, late and continuous breeding, as well as excessive and injudicious feeding and milking, are all frequent predisposing causes, and should be avoided.

With reference to the eradication of the disease in herds already affected, it is recommended that a thorough examination of the herd be made, using tuberculin test.

All animals found diseased should be slaughtered, and the remaining animals retested at intervals.

After the removal of these, attention should be paid, first of all, to the stables. Here, during the long confinement in the winter months, we may look for most of the inhalation disease so common in tuberculous cattle. Even when only a few cases of tuberculosis have been found, the stables should be disinfected by removal of all dirt and the subsequent application of disinfectants. Since tubercle bacilli are more resistant than most other disease germs, the strength of the disinfecting solution must not be less than as given. The following substances may be used:

(a) Corrosive sublimate (mercuric chloride), one ounce in about eight gallons of water (one-tenth per cent). The water should be kept in wooden tubs or barrels and the sublimate added to it. The whole must be allowed to stand for twenty-four hours, so as to give the sublimate an opportunity to become entirely dissolved. Since this solution is poisonous it should be kept covered up and well guarded. It may be applied with a broom or mop, and used freely in all parts of the stable. Since it loses its virtue in proportion to the amount of dirt present, all manure and other dirt should be first removed and the stable well cleaned before applying the disinfectant. After it has been applied the stable should be kept vacant as long as possible. Before the animals are allowed to return, it is best to flush those parts which the animals may reach with their tongues, to remove any remaining poison.

(b) Chloride of lime, five ounces to a gallon of water (4 per cent.). This should be applied in the same way.

(c) The following disinfectant is very serviceable. It is not poisonous, but quite corrosive, and care should be taken to protect the eyes and hands from accidental splashing:

Crude carbolic acid, one-half gallon.
Crude sulphuric acid, one-half gallon.

These two substances should be mixed in tubs or glass vessels. The sulphuric is very slowly added to the carbolic acid. During the mixing a large amount of heat is developed. The disinfecting power of the mixture is heightened if the amount of heat is kept down by placing the tub or glass demijohn, containing the carbolic acid, in cold water, while the sulphuric acid is being added. The resulting mixture is added to water in the ratio of one to twenty. One gallon of mixed acids will thus furnish twenty gallons of a strongly disinfectant solution having a slightly milky appearance.

(d) Whitewash is not in itself of sufficient strength to destroy tubercle bacilli, but by imprisoning and incrusting them on the wall of stables they are made harmless until destroyed by prolonged drying. Whitewashing should be preceded by thorough cleansing.

Particular attention should be paid to the sides and ceiling of stable. All dust and cobwebs should be periodically washed down. Those parts coming in contact with the heads of cattle, stanchions, halters, troughs, etc., should be frequently cleansed and disinfected, even when they have not been used by avowedly diseased cattle.

The thorough disinfection and renovation of all infected barns is imperative, and good drainage, light and ventilation should be secured.

Where these conditions cannot be obtained it would, in many cases, be more economical and satisfactory to build new stables, always observing the recommendations suggested for healthy herds.

Alex. Burr, M.D.V.,
Jas. B. Paige, D.V.S.,
John M. Parker, D.V.S.,
Committee on Tuberculosis

